# Role of Sensory Evaluation in Consumer Acceptance of Plant-Based Meat Analogs and Meat Extenders: A Scoping Review

**DOI:** 10.3390/foods9091334

**Published:** 2020-09-22

**Authors:** Martina Fiorentini, Amanda J. Kinchla, Alissa A. Nolden

**Affiliations:** Department of Food Science, University of Massachusetts Amherst, Amherst, MA 01003, USA; mfiorentini@umass.edu (M.F.); amanda.kinchla@foodsci.umass.edu (A.J.K.)

**Keywords:** sensory evaluation, consumer acceptance, descriptive analysis, meat analog, meat extender, plant-based, alternative protein, imitation meat

## Abstract

Growing demand for sustainable food has led to the development of meat analogs to satisfy flexitarians and conscious meat-eaters. Successful combinations of functional ingredients and processing methods result in the generation of meat-like sensory attributes, which are necessary to attract non-vegetarian consumers. Sensory science is a broader research field used to measure and interpret responses to product properties, which is not limited to consumer liking. Acceptance is evaluated through hedonic tests to assess the overall liking and degree of liking for individual sensory attributes. Descriptive analysis provides both qualitative and quantitative results of the product’s sensory profile. Here, original research papers are reviewed that evaluate sensory attributes of meat analogs and meat extenders through hedonic testing and/or descriptive analysis to demonstrate how these analytical approaches are important for consumer acceptance. Sensory evaluation combined with instrumental measures, such as texture and color, can be advantageous and help to improve the final product. Future applications of these methods might include integration of sensory tests during product development to better direct product processing and formulation. By conducting sensory evaluation, companies and researchers will learn valuable information regarding product attributes and overall liking that help to provide more widely accepted and sustainable foods.

## 1. Introduction

### 1.1. Background: The Need for Sustainable Alternatives to Meat

The meat industry is currently facing one of the biggest challenges of the past century: to meet the growing demand for animal products by providing high-quality protein without exceeding the critical limit of natural resources. Current predictions estimate that the world population will reach 9 billion people by 2050 [1] combined with the rising trend of meat consumption due to income increase in industrialized countries [2], which indicates that demand for animal-source foods is likely to double by 2050. This presents an alarming threat to our planet, as meat production is an intensive and unsustainable process, causing environmental problems such as deforestation, pollution, damage to hydrogeological reserves, and loss of biodiversity [3]. The livestock sector alone is responsible for 14.5% of human-made greenhouse gas emissions [4] and uses almost 30% of the world’s fresh water resources [5]. Another motivating factor is the issues surrounding animal welfare [6], with concerns regarding the unethical practices of factory farming as well as the excessive use of antibiotics used to fight new infections caused by potentially deadly pathogens.

Replacing meat with sustainable alternative proteins is one promising strategy to reduce meat consumption [7]. The environmental gains of relying on non-animal protein sources such as plants, insects, fungi, and algae, are significant. A complete switch to non-animal proteins in the human diet would reduce the use of natural resources currently dedicated to the livestock sector by 35–50% [8]. In Western countries, plant-derived proteins are more popular than other alternative proteins [9]. Soy products like tofu and tempeh, which originate from Asian countries, have been commercially available in the West since the 1960s and are now accepted by vegetarians and vegan consumers who avoid eating meat for ethical, environmental, or health reasons [10]. However, such products are not as popular among meat-eaters and flexitarians due to their low sensory appeal [11]. Many food companies have joined the alternative protein movement and promote sustainable eating by developing plant-based products with meat-like sensory attributes, often referred to as meat analogs, plant-based, or imitation meat. On a food processing level, recreating the texture and flavor of muscle meat starting from plant proteins has proved to be a challenge, often attributed to production of off-flavors typically by legumes and a lower saturated fat content that is responsible for tenderness and juiciness [12]. While there are many different processing methods to prepare meat analogs, one top-down strategy is high-moisture wet extrusion, which is highly successful in achieving a desirable structure, most resembling animal proteins [13]. Another strategy to achieve desirable texture and flavor while also reducing meat consumption is by partially replacing animal protein with plant-derived extenders. This is a common practice adopted by the food industry to improve the economical, functional, sustainability, and nutritional profile of processed meats [14].

Overconsumption of red meat in Western countries contributes to the development of cardiovascular disease due to the high saturated fat content [15]. This represents a major public health issue, specifically in the United States, where heart disease is the leading cause of death [16]. However, consumption trends observed in the last decade reveal that most Americans do not seem to be reducing their intake of red meat [2]. Identifying high-quality meat alternatives that mimic traditional meats may more effectively appease consumers without compromising the sensory qualities of meat products. Process optimization and new technologies aimed at utilizing novel plant-proteins are essential to the product development of meat analogs. Sensory evaluation, in the context of meat analogs, provides important information regarding the selection of processing methods and use of novel ingredients to achieve meat-like sensory attributes by providing both quantitative and qualitative data on taste, flavor, texture, and appearance.

### 1.2. Role of Sensory Evaluation in Consumer Acceptance of Meat Analogs

For meat analogs to successfully replace meat in the everyday diet, these novel products must be first accepted by the public in terms of overall liking. Sensory evaluation plays multiple roles in predicting consumer acceptance of meat analogs as this is not only influenced by the product’s sensory characteristics but also by person-related factors. These depend on the ethical aspects, political values, and ecological welfare involved in the production and can act as either drivers or barriers to acceptance of meat analogs. Data collected from a consumer survey in the U.K. and The Netherlands show that, while consumers are typically aware of the ethical and political implications of their food choices, purchase intention is ultimately driven by the product’s sensory attributes [11]. More specifically, the unfamiliarity with novel foods can alter expectations that may negatively impact sensory perception and overall liking [17]. To reduce consumer uncertainty to meat analogs, these are often marketed with slogans such as “tastes like meat” so that consumers can relate to their previous experience and form favorable expectations on the product’s performance. Sensory evaluation methods can gather data regarding consumers’ perceptions beyond the oral perception of foods. It is important to identify which product characteristics are drivers of product liking, while also taking into consideration differences between person-related factors. Integrating data of this kind with results from sensory evaluation and instrumental measurements provide a more accurate description of the physiochemical and sensory properties of meat analogs.

While the sensory properties of a food product play a collective role in forming positive expectations both before and during consumption, some may be more important than others. According to a 2019 survey of US adults, 86% of consumers considered taste to be the major driver of purchase intention [18]. In the same light, unpleasant or unexpected taste can represent a barrier to acceptance. In fact, non-vegetarians seem to be reluctant to try meat analogs due to the belief that consuming healthy products might compromise taste [19]. This obstacle can be overcome by developing products that meat-eaters will enjoy not only in terms of their individual sensory properties but also in the meal context in which they will be consumed. This includes other food components in the dish, such as rice, vegetables, and soups, as well as seasonings, spices, and sauces. A successful interaction of these ingredients depends on their sensory attributes. For instance, before consumption, shape, color, and appearance have a greater influence on consumer acceptance compared to flavor and texture [20]. This is because visual cues define the appropriateness of the meal, which is dictated by the cultural aspects of eating certain foods and by the individual preferences of the consumers. By contrast, consumers’ perception of flavor and texture of meat analogs are minimized to a certain extent, due to other ingredients in the meal that can have either a masking or enhancing effect. Sensory evaluation can help to increase consumer acceptance of meat analogs by investigating the complex interaction between factors that are known to affect meal appropriateness with the goal to understand the best way to market these products based on their sensory properties.

### 1.3. Sensory Evaluation Methods

This narrative review focuses on two main categories of sensory evaluation that are summarized in Table 1. Consumer acceptability tests, also called hedonic of affective tests, assess the degree of liking of a product based on its sensory appeal. Untrained participants perform the test, usually greater than 100 participants, who are screened for product usage [21]. A common way to assess acceptability is through hedonic scales where the participants indicate how much they like or dislike the sample in terms of a specific sensory property, such as appearance, flavor, taste, and texture, and can also include overall liking/acceptance. The most commonly used scale is the 9-point hedonic scale that ranges from “like extremely” to “dislike extremely” [21]. Other scales include the visual analog scale (VAS), a non-marked, anchored line, and the “just about right” (JAR) scale, which is used to adjust the proportions of certain ingredients that can alter the intensity of a sensory characteristic (e.g., spiciness, saltiness). A set of check-all-that-apply (CATA) terms can also be used to collect hedonic responses. This is a format in which respondents are presented with a list of terms and asked to select all those that apply to each sample. The list of terms can be either generated by a group of trained panelists or it can be derived from the available literature. In other instances, the CATA method can be used to estimate the intensity of a specific attribute by examining the frequency in which the attribute is experienced; however, in the current review, the study utilizing this method has selected terms that are hedonic in nature and, therefore, grouped with acceptability tests. Descriptive sensory analysis provides a more detailed assessment of the product’s sensory profile. It determines both a qualitative and quantitative measurement of the intensities of each sensory attribute. Descriptive analysis techniques include the Flavor Profile^®^, Quantitative Descriptive Analysis^®^, Texture Profile^®^, and Sensory Spectrum^®^ [21]. Trained panelists, often 8–12, undergo extensive training on the relevant attributes [21]. Following training, panelists independently rate intensity of each attribute. These methods provide different information regarding the sensory profile of the product. Consumer data identifies which sensory attributes are needed to increase overall liking, whereas data from descriptive analysis is more accurately quantified and can significantly contribute to the direction of product development. An appropriate selection of the method is important for obtaining the desired sensory information to improve the final product.

### 1.4. Organization and Scope of the Review

The application of consumer studies and descriptive analysis provides useful information about the sensory profile and consumer acceptance of foods and beverages. To the authors’ knowledge, there has not been a literature review on the application of these sensory evaluation methods to meat analogs and extended meat products. This narrative review summarizes the literature evaluating the sensory attributes of meat analogs and meat extenders. Specifically, it focuses on studies that involve consumers’ evaluation of products that uses hedonic and/or descriptive analysis methods. Here, the review focuses on plant-based products as these are the most commercially available and are preferred by consumers [22,23], rather than other alternative protein sources (e.g., insect, fungi, and algae). Moreover, the review includes studies of extended meat products where partial replacement of meat protein was at least 30% following the analysis of consumer data revealing a preference for hybrid products with a 50:50 ratio of plant-based to meat ingredients [24]. By reviewing the available literature, the goal is to show the advantage of evaluating the sensory properties of meat analogs to predict consumer acceptance by understanding the factors that affect hedonic preference. The purpose of this review is to summarize the changes that occur in sensory attributes resulting from the integration and innovation of processing techniques of novel plant-proteins. There is an opportunity to build a greater understanding of the impact of novel plant-proteins and processing technology on the taste, flavor, and texture profile. Achieving desirable meat-like qualities will help to increase consumers’ acceptance with the long-term goal of reducing the consumption and production of animal livestock that improves human health and environmental sustainability.

## 2. Search Criteria Methods

Articles were searched from Web of Science and Google Scholar using keywords and restriction on publication year from 2000 to 2020. Products of interest were searched using “meat analog*”, “meat substitutes”, “alternative protein”, “plant-based”, “hybrid meat”, “meat extenders”, “meat replacement”, and “extrusion”. Consumer studies and sensory descriptive analysis methods were selected using “consumer liking”, “consumer acceptance”, “consumer perception”, “sensory quality”, “sensory characteristics”, “sensory properties”, and “descriptive sensory evaluation”. Studies involving meat analogs made with insects, mycoproteins, algae, and in vitro meat as a protein source were removed. In the case of meat extenders, only studies evaluating products in which at least 30% of the protein content was replaced with plant proteins were selected. Studies with products containing functional ingredients as food additives but where animal protein was not replaced with plant proteins were excluded. While numerous studies on consumer perception of meat analogs were found, online surveys, questionnaires, and focus groups where data were collected based on visual or verbal information and not through tasting were excluded. Following an initial search, and secondary screening of the above criteria, the review resulted in the selection of 14 articles.

## 3. Literature Review

Fourteen research papers were found within the defined search query. These are summarized in Table 2. Of the 14 selected papers, 11 evaluated consumer acceptance with hedonic testing and 3 used sensory descriptive analysis. Eleven evaluated 100% plant-based meat analogs, and 3 evaluated extended meat products. Twelve evaluated the addition of ingredients and 2 evaluating processing/cooking methods. The main protein source was soy in the form of isolate, concentrate, or flour, followed by wheat gluten and peanut. All meat analogs were prepared by either extrusion processed, emulsified systems (e.g., sausage) or formed materials (e.g., nuggets, meatballs, patties). Samples were cooked in different methods (e.g., oven-baked, pan-fried) with or without seasonings or marinades, depending on what type of processed meat the product was meant to recreate. During sensory evaluation, the control samples consisted of either a commercial meat analog or a meat equivalent product. For studies testing the most adequate concentration of functional ingredients, the control sample was the one where the ingredient of interest was not added. Table 3 summarizes the reviewed articles, which are categorized by the sensory attribute of interest, by highlighting the strategies that have been tested, the type of control used, and the main finding. Table 2 shows a summary of articles employing consumer and descriptive analysis tests.

### 3.1. Color and Overall Appearance

The overall appearance of a product is important for priming consumers and developing expectations prior to consumption. A disconfirmation of expectations occurs when the perceived liking after consumption is below the expected liking, which may occur when the visual cues misrepresent the taste, odor, and flavor of the product [39]. Thus, it is important to deliver high-quality sensory attributes that are perceived both before and during consumption. The overall appearance of meat analogs should resemble familiar meat products in order to set positive expectations. A combination of cooking parameters, such as time and temperature, have been tested to improve the overall appearance of meat analogs as they can impact the final visual appearance of the cooked product. Gomez and colleagues [28] tested the effect of changing cooking time and temperature on the color attributes of a ready-to-eat soy meat analog using the *sous vide* technique, which consists of cooking a vacuum-sealed product at low temperatures in a water bath. Both the meat analog and a beef equivalent were treated with two marinades, beer and teriyaki, and cooked at varying times and temperatures. The main ingredient in teriyaki marinade was pineapple juice (71%) resulting in a light-yellow color, whereas the beer marinade was made with pale lager beer (80%), resulting in a more golden color. A hedonic test was performed by 73 consumers who rated three visual parameters of the product. No significant difference in hedonic scores was detected between the samples, suggesting that the meat analogs were equally accepted as the beef samples in terms of visual appearance. In addition, results from color analysis revealed that both samples cooked with similar parameters had the same values for lightness and redness, which is the characteristic color parameter for meat products, suggesting that this cooking technique can be used to develop meat analogs with a similar appearance as their meat equivalent, regardless of the type of marinade used. Instrumental color analysis also revealed higher yellowness values in the samples cooked with teriyaki marinade compared to the beer marinade. This was attributed to the lighter yellow color of the teriyaki marinade. These results can be used to direct product development of meat analogs in terms of color depending on the desired outcome.

Certain ingredients can affect the color and appearance of meat analogs. Sharima-Abdullah and colleagues [29] developed meatless nuggets by changing the ratio of chickpea flour to texturized vegetable protein. Hedonic test showed that color and appearance scores increased as chickpea flour concentration increased. These results were explained by the presence of carotenoids in chickpea contributing to a yellow color, which was appealing to the participants. Surprisingly, increasing hedonic scores for color did not correlate with increasing overall acceptance scores. In fact, overall acceptance seemed to decrease as the percentage of chickpea flour increased. A 10:30 ratio chickpea flour to textured vegetable protein (TVP) resulted in the highest acceptance scores. This was explained by an increase in dislike of the nuggets in terms of taste. This provides evidence that multiple sensory attributes play an important role in consumer acceptance.

One processing limitation of using plant proteins is that the color of meat analogs may fade out when exposed to light or oxygen, leading to an unappetizing product [40]. Marinating can be used as a preparation method to change the color of meat analogs prior to cooking. Other ingredients used in the formulation of meat analogs can dictate the color of the final product. Teriyaki and/or beer marinades as well as chickpea flour are acceptable ingredients to obtain a bright yellow color that is appealing to consumers. Cooking parameters such as time and temperature can also affect the appearance of meat analogs. A higher moisture content in a meat analog cooked at high temperatures can lead to deeper penetration of light in the product, resulting in a brighter color. These studies demonstrate that several approaches can impact consumer ratings for the color and visual appearance of meat analogs.

### 3.2. Taste, Flavor, Aroma

A common disadvantage of using plant proteins in meat analogs is the generation of volatile compounds from the lipid oxidation of unsaturated fatty acids that contribute to the formation of unappealing odors and flavors [41]. To overcome this problem, food scientists develop recipes that include flavoring mixtures with seasonings, spices, and enhancers that can both replicate the typical flavor of smoked meat as well as mask the beany, grassy, or green aroma of pulses. To assess consumer acceptability of meat analogs in terms of taste, flavor, and aroma, the sample is presented in a way so that it resembles the equivalent meat product. In a study performed by Rehrah et al. [30], three formulations of peanut-based minced product were evaluated against a commercial soy-based minced product in a seasoned puff pastry application. The peanut-based meat analog was made by fortifying defatted peanut flour with peanut protein concentrate. The mixture was extruded, ground into a beef-like mince, and stuffed into a rolled puff pastry to provide a more realistic version of a commercial snack. All three formulations of textured peanut protein concentrate (PPC) were seasoned with beef flavor and soy sauce as a flavor enhancer. In addition, the first sample contained tomato powder, the second sample contained crushed red pepper, and the third sample had no modifications. A sensory panel of 60 participants rated the peanut-based sample along with the commercial soy-based control in terms of beefy flavor, off-flavor, and spiciness on a 9-point hedonic scale. Participants were also asked to determine spiciness on a just-right scale with three intensities (too little, just right, too much). Of the three formulations of peanut-based meat analog, the one containing crushed red peppers had the most acceptable meaty flavor, the least dislike of off-flavor, and the most adequate spiciness level. These results suggest that the addition of flavors, enhancers, and spices can positively affect consumer acceptance of taste of a meat analog. However, while PPC performed better than soy-based formulations in a puff pastry application, the study did not include comparisons against a traditional meat formulation, suggesting that the choice of the control is a significant variable to be considered. On one hand, if the reference and test samples consist of different plant proteins, hedonic responses may be affected by the additional spices, with the most seasoned formulation resulting in higher acceptance scores. Alternatively, using a full-meat sample as a control would help to best determine how a meat analog compares to the desirable sensory properties of a traditional animal product.

Comparing plant-based meat analogs to their meat equivalent can be adopted as a strategy during evaluation of extended meat products. Wong and colleagues [31] developed three formulations of hybrid beef patties by substituting 10%, 20%, and 30% ground beef with hydrated textured soy protein (TSP). A first hedonic test with 55 consumers showed no significant difference in overall liking scores for all formulations compared to the all-beef control. In a second hedonic test, 56 consumers evaluated the sensory properties of a hybrid beef patty with 20% TSP substitution and all-beef patty both with reduced sodium level. Liking scores for flavor were slightly lower in both the 20% TSP patty and the all-beef patty with reduced sodium compared to the all-beef control with regular sodium level. This suggests that substitution of beef with plant protein up to 30% can lead to acceptable liking scores as long as the sodium content remains unchanged. These findings reveal that maintaining a high sodium level in meat analogs is important for consumer acceptance in terms of flavor, although this may lower the nutritional quality of the final product. In another study, Grasso et al. [37] developed four types of hybrid meatballs by substituting 15% and 30% of beef with TSP in duplicates, with or without nutritional yeast, which was used as a flavor enhancer for its strong umami flavor. Sixty participants evaluated the four samples and an all-beef control by assessing degree of liking on a 9-point scale in terms of flavor, texture, and overall acceptance. In addition, participant used the check-all-that-apply (CATA) method by selecting the most appropriate terms to describe the samples. This method provides a complete description of the sensory characteristics of the samples. A list of 24 terms was chosen from the available literature on meat products. The CATA terms related to flavor were “tasty’’, ‘‘bland’’, ‘‘cheesy’’, ‘‘weak meaty’’, ‘‘strong meaty’’, ‘‘wheat-cereal like’’, ‘‘unusual’’, and ‘‘characteristic’’. Results from the hedonic test showed that addition of 15% TSP and nutritional yeast resulted in the highest liking scores for flavor and overall acceptance. Results from CATA analysis revealed that this sample was most associated with the term “tasty” and less associated with “bland”, while the 30% TSP without yeast was most associated with “wheat-cereal like”, suggesting that the absence of flavor enhancers in a sample with a high percentage of soy content may lead to the detection of strong off-flavors. Interestingly, the all-beef control was most frequently associated with the term “bland”. This suggests that the selection of control is important in understanding and interpreting consumer acceptance of hybrid products. It is important to select a control that is liked by consumers and is a good representation of the target product, as a low-quality control product could lead to misinterpreting results. Partial replacement of animal protein with plant protein provides the opportunity to improve the sustainability of meat products while also improving the nutritional profile of processed meat. However, addition of plant proteins might affect the overall product quality. The addition of up to 15% vegetable protein is appropriate to improve healthfulness of meat products without reducing the quality of sensory attributes.

When the objective of the study is to test how different concentrations of a flavoring agent affect the sensory attributes of the final product, the sample without the added ingredient is used as a control, as opposed to using a full-meat product or a commercial meat substitute. Chiang et al. [32] added Maillard-reacted beef bone hydrolysate (MRP) at four concentrations to a meat analog made with soy protein concentrate and wheat gluten to improve its sensory attributes. Beef-bone extract, a by-product of meat processing, can be used as a flavor-enhancing agent by undergoing enzymatic hydrolysis to increase the proportion of free amino acids, followed by Maillard reaction through addition of reducing sugars to produce heterocyclic compounds. These molecules contribute to the typical flavor and aroma of smoked meat when this is cooked on the grill. Sensory evaluation by a group of 55 consumers revealed that 20% MRP was the optimal level for acceptance, resulting in the highest sensory scores for meaty aroma and meaty taste. By contrast, addition of 40% MRP received the lowest scores in all attributes due to bitter taste and a burnt appearance, while 0% MRP (the control) resulted in a weaker meaty taste and an undesirable pale brown color. The addition of MRP, under a certain concentration, helps to increase desirable “meat” flavors and increases acceptance compared to unflavored meat analog. It is not known if the addition of MRP would compare to a full-meat control. However, this product includes meat extracts, making it inappropriate for vegans and vegetarians; yet, it demonstrates the use of hydrolyzed protein materials can enhance desirable flavor attributes, which is important in increasing overall acceptance of meat analogs.

Katayama and Wilson [25] used a similar approach in their study. The aim was to determine the most acceptable concentration of vegetable-based “chicken” and “shrimp” flavors added to textured soy meat analogs prepared in four different shapes (narrow strip, wide strip, shred, and bit) and with two cooking methods (fried and baked). The use of vegetable-based flavors provides an acceptable alternative to meat-by products like beef-bone extract, which may represent a barrier for vegan consumers. In the study, “chicken” flavor was added in either powder or liquid form at 3% and 4% to all four shapes of extrudates, which were fried, while two types of “shrimp” flavor, one oyster-like, the other a combination of oyster and crab-like, were added to shred-shaped extrudates, which were baked. A trained sensory panel of 14 participants generated a list of descriptive terms based on chicken and shrimp flavors to evaluate the samples. All formulations were rated on an analog scale using unflavored samples as controls. Results showed that the presence of 4% flavoring enhanced the overall saltiness and meatiness. The size of the product sample appeared to significantly impact the “chicken” flavor in powder form as the narrow strip-shape was more intense in the oily flavor compared to the wide strip sample. This was attributed to the formation of air pockets in the former, responsible for encapsulating flavor molecules during frying. Following descriptive analysis, researchers conducted a consumer preference test with 125 volunteers for evaluating the “chicken” flavored product. Consumer results revealed that the chicken-flavored sample was most accepted when fried rather than baked. However, chemical analysis showed that the fried samples had more than 3 times higher fat content than the baked samples, suggesting that the higher fat content may increase liking. Moreover, this cooking method can negatively impact the nutritional quality of the product. In this study, researchers first used a descriptive sensory analysis to determine the best product formulation based on variables, such as type and concentration of the flavoring agent as well as shape and cooking method of the sample. Then, a consumer preference test was performed to evaluate the product based on a single variable. Performing a consumer test following descriptive analysis is a common strategy to efficiently assess consumer acceptance by combining both quantitative and qualitative data on the sensory profile of meat analogs in order to collect more specific information that can be used to apply changes in the recipe or processing method.

The formation of unappealing odors and flavors during processing of plant proteins represents a barrier to acceptance of meat analogs. Addition of spices, seasonings, and flavor enhancers is an appropriate strategy to mask the beany and grassy aroma, specifically in pulses. The nature of the added ingredient may need to comply with vegetarian and/or vegan consumers’ dietary restrictions. Meat by-products such as beef bone hydrolysate can provide a “meaty” flavor to meat analogs in order to increase acceptance among non-vegetarian consumers. Yet, vegetable-based mixtures have been successful in recreating the flavor of poultry or seafood products. Use of spices, such as red pepper flakes, can increase spiciness to overcome off-flavors, while nutritional yeast can be used as an enhancer to provide a “meaty” umami taste. Sensory evaluation methods aimed at identifying acceptable flavoring ingredients can be influenced by the selection of the control product. Comparing two different kinds of plant proteins as opposed to using a full-meat control can impact conclusions drawn regarding product liking. Further research should focus on evaluating the taste and flavor of meat analogs in a meal context, as additional foods in the dish can alter the perception of oral sensation of meat analogs.

### 3.3. Texture

Another challenge for meat analogs is the recreation of the unique texture, mouthfeel, and juiciness of traditional meat products [41]. For meat analogs, the focus has been on the selection of plant protein to recreate the physiochemical properties of animal protein. Factors include the ability to encapsulate fat, their oil- and water- holding capacity, and gelling and emulsifying properties, which can be measured through texture analysis. Instrumentation combined with sensory evaluation, such as consumer liking, can be a helpful indicator of consumer acceptance for texture.

Choosing the right protein source is essential to develop vegetarian versions of meat products. Gluten is the main protein source in wheat, and it is commonly added to processed meats as a binding agent for its viscoelastic properties that allow to form a cohesive network in the product. Kamani and colleagues [38] used soy protein isolate and wheat gluten to develop two products, (1) a meat-free sausage and (2) a reduced-meat sausage containing only 20% of chicken. Results from the hedonic test showed no significant differences in the liking texture scores between samples containing both 80% and 100% plant proteins compared to the full-meat control. This was associated with texture analysis data, showing a reduced cooking loss and a better emulsion stability in the samples with partial and total replacement of meat. Implementing the results from sensory evaluation, which is subjective, with instrumental results from texture analysis allows to either confirm the outcome of the study or identify possible inconsistencies in the methods. However, it should be noted that Kamani and colleagues [38] collected hedonic responses using a trained panel, which goes against the standard procedure of sensory evaluation method for consumer acceptability. Thus, these results should be analyzed with caution due to methodological issues and represent a limitation of the study.

In addition to the selection of plant protein source, another way to improve the texture of meat analogs is by using food additives. Hydrocolloids have gelling, thickening, emulsifying, and stabilizing properties due to their ability to interact with water, proteins, starch, and other components in the food product. The meat industry often incorporates hydrocolloids in meat sausages to compensate for textural quality loss that occurs when part of the fat and salt is reduced. Common types of hydrocolloids for meat analogs include carrageenan, an algae-derived polysaccharide, xanthan gum, a polysaccharide produced by bacterial fermentation, and konjac mannan, a tuber-derived heteropolysaccharide. Majzoobi and colleagues [33] found that addition of either 0.3–0.6% kappa-carrageen or 0.6% konjac mannan resulted in the highest consumer acceptability scores of a soy protein isolate (SPI) sausage. These results were confirmed by textural analysis showing that sausages produced by k-carrageenan and konjac mannan had the highest water-holding capacity, leading to the production of a strong network within the sausage matrix and an increase in tenderness of the samples. Similarly, Palanisamy et al. [34] found an improvement in textural attributes of a soy meat analog by increasing the concentration of iota-carrageenan (ICGN), with 1.5% being the optimal level for hedonic texture ratings. However, results showed that all test samples had poor overall acceptability, including the 0% ICGN control sample, as no samples included seasonings or spices, which was intentional to avoid any influence on the perception of texture. In this instance, while texture was improved, creating a desirable texture alone is not sufficient to create an overall suitable product. It is important to consider how sensory attributes together influence consumer acceptance. Moreover, it should be noted that the consumer group used in this study is small, making it inappropriate to generalize the hedonic responses regarding the product sample.

Other functional ingredients that are used as food additives to improve the texture of meat analogs include thickeners and emulsifiers. Bleached tomato pomace, a by-product of tomato processing, is rich in fiber and pectin and is used as a thickening agent. Savadkoohi et al. [35] developed three sausage formulations, namely soy, beef, and ham, which were evaluated by a descriptive sensory analysis based on the added concentration of bleached tomato pomace. Three commercial samples with no tomato paste addition were used as controls. Thirty consumers rated the liking of sensory properties on a line scale for each sample. Sensory scores showed that addition of bleached tomato pomace at 5% was the optimal level for acceptance. This was confirmed by instrumental textural analysis showing that addition of tomato pomace increased textural hardness and chewiness of the meat analog. However, analysis revealed that additive concentration greater than 5% resulted in an undesirable orange-green color compared to the control. In another study, Wi et al. [26] added non-animal-based liquid additives, including water, hydrated SPI, canola oil, and lecithin to an emulsified meat analog made with TVP and SPI. Sensory evaluation was performed by 10 panelists who rated the intensity of firmness, elasticity, stickiness, compactness, roughness, soy taste, oil taste, juiciness, and overall acceptance on a 7-point scale. Results showed that juiciness was positively affected by water treatment, whereas overall acceptance was positively correlated with emulsion treatment.

Finally, the processing methods can influence texture and mouthfeel properties of meat analogs. Lin et al. [27] tested the effects of different moisture content and cooking temperature on the attributes of an extruded meat analog made with soy protein isolate. For sensory data, a trained panel of 9 judges evaluated the samples based on 7 descriptive terms related to mouthfeel. The authors combined data from sensory evaluation and instrumental analysis, including texture profile analysis, water absorption capability, and microstructure, which was determined by scanning electron microscopy. They found that changes in moisture content had a greater effect on sensory and physiochemical properties than cooking temperature. However, while results showed a correlation between directional structure and textural attributes like hardness or chewiness, the study did not determine whether this affected consumer acceptance. In another study, Yuliarti et al. [36] developed plant-based nuggets using a freeze structuring technique, which consists in the freezing of a protein emulsion to generate a unique fibrous structure and a subsequent removal of ice crystals to generate a porous and fibrous microstructure, similar to that of animal meat. Five formulations of nuggets were developed by changing the ratio of pea protein to wheat protein. Two of the five samples were used as controls, namely 17:0 and 0:17 pea protein isolate (PPI) to wheat protein (WP). A hedonic test was conducted with 42 untrained participants who rated the analog in terms of texture on a 5-point acceptance scale. Results showed that freeze structuring technique was able to form a fibrous and layered structure of the plant-based protein nuggets, however, this technique was also dependent on the type of protein used. In fact, a 4:13 PPI to WP ratio was the most preferred analog compared to controls. Microstructure of this analog indicated fibrous and layered structure, while textural profile analysis was found to be related to the viscoelastic properties of WP and was strongly affected by the extent of cross-linking between protein molecules. A combination of hedonic tests, descriptive analysis, and analytical methods is ideal to explain the impact of processing parameters on texture and mouthfeel attributes.

The structure of muscle meat is challenging to recreate without the use of animal proteins. The selection of plant proteins with viscoelastic properties like wheat gluten can help to improve the texture and mouthfeel of the final product. In addition, ingredients like hydrocolloids, gels, and gums can be used for their emulsifying properties. Optimizing synergistic effects of different ingredient ratios can further advance texture quality perception. Extrusion parameters can be modified to obtain a desired texture, with changes in moisture content playing a greater role in physiochemical properties than cooking temperature. Freeze structuring technique can generate a fibrous and layered structure in plant-based nuggets. This strategy can be adopted when extrusion cooking is not available. Future sensory evaluation studies should explore how the texture of meat analogs is affected by other components in the meal with different consistencies, such as sauces and soups, which can alter the perception of oral sensations of meat analogs. Moreover, the application of commercial ingredients that utilize the synergistic effects of soy and gluten should be further investigated to optimize the creation of desirable textural properties of meat analogs.

## 4. Conclusions

This review focused on research papers evaluating the impact of ingredients and processing methods for meat analogs and meat extenders on sensory attributes and consumer acceptance. This area of research helps provide important information on the use of novel plant proteins in meat analogs, as they are known to have processing limitations. The color of plant-based products may fade out due to light or oxygen exposure, leading to an unappetizing appearance. An undesirable taste can occur due to off-flavors from lipid oxidation of unsaturated fatty acids in plant protein ingredients and products. Texture attributes such as fibrous structure, tenderness, and juiciness of muscle protein are very challenging to recreate in plant proteins due to the reduced saturated fat content. Current research in food science is investigating strategies to improve the overall quality of meat analogs. In terms of increasing consumer acceptance, studies have focused on adjusting formulations and cooking methods to improve color, flavor, and texture of meat analogs. In this regard, it should be noted that the choice of protein source is an important factor to be considered in the development of meat analogs as it can influence the perceived sensory attributes of the finished product. For instance, leguminous proteins such as soy and pea, while high in protein content, have some processing limitations leading to the production of strong off-flavors. Peanut protein has some processing advantages as it is very nutrient dense due to the high protein, fat, and fiber content. Moreover, peanut protein was found to perform better than soy in a puff pastry application in this review. Wheat gluten can be used for its viscoelastic properties to improve the texture of meat analogs, however, it has a much lower protein content than soy and peanut. Finally, soy, peanut, and wheat are three major food allergens, suggesting that the application of any of these ingredients may lower consumer acceptability of meat analogs depending on the consumer.

Sensory evaluation methods, involving untrained or trained consumers, can provide a better understanding of how different factors, such as processing and ingredients, affect quality attributes and overall consumer acceptance of meat analogs. Studies that employed more than one sensory method were able to identify the combination of parameters or ingredients that resulted in the highest acceptance scores for the sensory attribute of interest. Studies that used a qualitative approach to evaluate the samples were useful to identify the magnitude in which sensory attributes were influenced by test parameters. However, using this approach alone reduced the ability to understand the potential to impact or improve consumer acceptance values. A combination of hedonic testing and descriptive analysis provides a more holistic understanding and an ideal approach to evaluate the sensory profile of meat analogs while also being able to identify the strategies to increase consumer acceptance of these novel foods. Moreover, it is important to follow standardized procedures when choosing the appropriate sensory evaluation method, as these might compromise the reliability of scientific results. Factors such as the number of participants and training can influence the sensory scores and lead to inaccurate interpretation.

Sensory characteristics, including color, flavor, and mouthfeel of meat analogs can be modified through addition of functional ingredients and selection of processing methods. For color parameters, marinades, or other ingredients containing yellow pigments can be used to improve the overall appearance of the final product. Different flavoring agents and seasonings can either mask the undesirable odors of plant proteins or recreate the umami and “meaty” flavor. Texture can be adjusted to resemble muscle structure through the application of plant proteins as independent materials and as blends, with or without the addition of hydrocolloids to change the viscosity. Extrusion cooking or freeze structuring technique results in desirable fibrous structures. Sensory data is a key component in understanding the physiochemical characteristics of novel plant proteins to increase consumer acceptance of meat analogs in order to make a significant advancement in more sustainable and healthy foods.

## Figures and Tables

**Table 1:** Summary of sensory evaluation methods used to evaluate plant-based meat analogs.

Consumer Acceptability Test	Descriptive Analysis Test
Assesses degree of liking of a product based on its sensory appeal	Provides a detailed assessment of the product’s sensory profile
Uses 100 or more participants with no previous training	Uses 8–12 trained panelists
Hedonic responses are collected through 9-point hedonic scales, visual analog scales, just about right scales, or CATA questions	Sensory scores are collected through intensity scales for each attribute of interest

**Table 2:** Summary of articles employing consumer and descriptive analysis tests.

Sample	Protein Source	Target Model	Participants	Country	Reference
Descriptive analysis studies					
Meat analog	TSP	Extrudate	14	USA	Katayama and Wilson [25]
	TVP, SPI	Emulsified product	10	Korea	Wi et al. [26]
	SPI	Extrudate	9	USA	Lin et al. [27]
Consumer and hedonic studies					
Meat analog	SF	Beef fillet	73	Spain	Gómez et al. [28]
	TSP	Chicken nugget	110	Malaysia	Sharima-Abdullah et al. [29]
	DPF, PPC	Beef-like mince	60	USA	Rehrah et al. [30]
	TSP	Beef patty	55/56	USA	Wong et al. [31]
	SPC, WG	Beef-like mince	55	New Zealand	Chiang et al. [32]
	TSP	Extrudate	125	USA	Katayama and Wilson [25]
	SPI, TSP	Meat-free sausage	24	Iran	Majzoobi et al. [33]
	SPC	Extrudate	18/17	Germany	Palanisamy et al. [34]
	SPI	Meat-free sausage	30	Iran	Savadkoohi et al. [35]
	PPI, WP	Chicken nugget	42	Singapore	Yuliarti et al. [36]
Extended meat product	TSP	Meatball	60	UK	Grasso et al. [37]
	SPI, WG	Meat-free sausage	8 (trained)	India	Kamani et al. [38]

DPF: defatted peanut flour; PPC: peanut protein concentrate; PPI: pea protein isolate; SF: soy flour; SPC: soy protein concentrate; SPI: soy protein isolate; TSP: textured soy protein; TVP: textured soy protein; WG: wheat gluten; WP: wheat protein.

**Table 3:** Summary of findings for reviewed articles.

Sensory Attributes	Approach	Control	Findings	References
Color, appearance	Combination of *sous vide* cooking parameters for an RTE soy meat analog: time (90, 120, 150 min), temperature (70°, 80 °C) and marinade (teriyaki and beer)	RTE beef	For each combination of cooking time and temperature, both the RTE meat analog and beef sample resulted in similar lightness, redness, and color intensity regardless of the marinade type	Gómez et al. [28]
	Changing ratios of chickpea flour to TVP: 30:10, 25:15, 20:20, 15:25, 10:30	Commercial chicken nuggets	A 10:30 chickpea flour to TVP ratio resulted in the highest acceptance scores	Sharima-Abdullah et al. [29]
Taste, flavor, aroma	Addition of seasonings and spices to a PPC meat analog	Commercial soy meat analog	The highest level of spices and crushed red peppers had the most acceptable meaty flavor, the least amount of off-flavor, and the most adequate spiciness level	Rehrah et al. [30]
	Sodium reduction from 1.5% to 1.1% in three hybrid TSP/beef patty formulations with 10%, 20%, 30% TSP substitution	100% beef patty with 1.5% sodium	Substitution of beef with TSP up to 30% resulted in similar acceptability scores to the control. Sodium reduction resulted in slightly lower acceptability scores compared to control	Wong et al. [31]
	Addition of nutritional yeast to a TSP hybrid meatball	100% beef meatball	15% TSP with yeast received the highest flavor and overall acceptability scores, was most associated with the term “tasty” and less associated with “bland”	Grasso et al. [37]
	Addition of MRP at 10%, 20%, 30%, 40% to a soy meat analog	0% MRP	20% MRP resulted in the highest sensory scores for meaty aroma and meaty taste	Chiang et al. [32]
	Addition of vegetable-based “chicken” or “shrimp” flavor at 3% and 4% to four shapes of soy meat analogs prepared with two cooking methods (fried or baked)	Unflavored sample	Highest flavor concentration with frying method received higher scores in terms of flavor intensity and saltiness	Katayama and Wilson [25]
Texture	Addition of SPI and WG at 80%, 100% to a chicken sausage	100% chicken	Samples with partial and total replacement of meat with plant proteins received higher liking scores for texture due to reduced cooking loss and better emulsion stability	Kamani et al. [38]
	Addition of j-carrageenan, konjac mannan and xanthan gum at 0.3%, 0.6%, 1.0%, 1.5% to an SPI sausage	0% hydrocolloids	0.3–0.6% kappa-carrageen or 0.6% konjac mannan resulted in highest acceptability scores	Majzoobi et al. [33]
	Addition of ICGN at 0.75%, 1.5%, 2.25%, 3% to a soy meat analog	0% ICGN	1.5% ICGN was the optimal level for acceptance of texture	Palanisamy et al. [34]
	Addition of bleached tomato pomace at 1%, 3%, 5%, 7% to an SPI meat-free sausage, a beef frankfurter and beef ham	0% bleached tomato pomace	3% and 5% bleached tomato pomace in meat-free sausage resulted in the highest scores for juiciness	Savadkoohi et al. [35]
	Addition of non-animal based liquid ingredients at different concentration ranging 15–35%	N/A	Water treatment affected juiciness more than the oil treatment	Wi et al. [26]
	Extrusion of a soy meat analog with moisture content at 60%, 65%, and 70% and cooking temperature at 138, 149, and 160 °C	N/A	Moisture content had a greater effect on sensory attributes than cooking temperature	Lin et al. [27]
	Changing ratios of PPI to WP: 7:0; 13:4; 8.5:8.5; 4:13, 0:17	Commercial 100% PPI and 100% WP meat analogs	A 4:13 PPI to WP ratio resulted in highest acceptance scores	Yuliarti et al. [36]
